# Everything Is Science: A Free City-Wide Science Festival

**DOI:** 10.3389/fcomm.2020.00068

**Published:** 2020-09-02

**Authors:** Jarrod W. Creameans, Michelle G. Pitts, Olivia White, Kellen M. Greenwell, Kristie Colón, Sylvie Garneau-Tsodikova, Vincent J. Venditto

**Affiliations:** College of Pharmacy, University of Kentucky, Lexington, KY, United States

**Keywords:** public engagement, public health, science communication, science literacy, STEM education

## Abstract

A week-long, city-wide science festival called *Everything is Science *(*EiS*) was developed to educate the community in an informal manner. The festival serves as a platform for presenters from diverse professions to give engaging talks (without PowerPoint slides) to the public, free of charge, in restaurants and bars around town. Over 350 people attended the events over 5 days with 33 presenters. Surveys completed by attendees and session coordinators indicate strong support for this festival. Altogether, the *EiS* festival serves as a no-cost method to engage with the community and improve science literacy with potential for adoption in other cities.

## INTRODUCTION

As the public is presented with misleading or inaccurate information and an increasing knowledge gap between scientists and non-scientists, unique strategies to engage communities around science are needed (Basiliko and Gupta, 2015; Science outreach in the post-truth age, 2017; Roche et al., 2020). The benefits of public outreach have been established and innovative strategies are necessary to effectively harness these societal benefits (Banerjee, 2016; Clark et al., 2016; Committee on Science Literacy Public Perception of Science, 2016). While the benefits of science communication are well-established, the strategies to engage non-scientists have evolved over time (Bucchi and Trench, 2008). These changes have shifted science from a top-down approach where the scientist teaches the non-scientist in what is considered a knowledge-deficit model to a platform where the public elevates science through citizen-science programs (National Academies of Sciences, Engineering, and Medicine, 2018). This paradigm shift has positioned science festivals to involve more dialogue than top-down education, which has been demonstrated by Science Cafés^1^, Café Scientifique^2^ and Nerd Nite (Tan and Perucho, 2018), among others. The popularity of these festivals and others like it surged after 2009 when the US National Science Foundation (NSF) established the Science Festival Alliance (SFA) providing a collaborative network of individuals and organizations interested in promoting science in their communities (Durant, 2013). The SFA has also standardized festival evaluation through EvalFest^3^, which utilizes crowd-sourced data to analyze and validate mechanisms to measure festival impact.

While the infrastructure for such events are bolstered by federal funding and a consortium of like-minded individuals and organizations, many communities still lack science outreach programs that would provide informal enrichment for the community. Oftentimes, community outreach is intimately linked with extramural funding or relies on substantial fundraising to achieve their goals. However, strategies that uncouple funding from outreach activities have the capacity to become sustainable outside of standard funding mechanisms. Furthermore, as high school graduates nationwide continue to struggle with undergraduate preparedness in science, technology, engineering, arts, and math (STEAM) (Brown et al., 2016), public outreach is a critical mechanism to provide educational enrichment and establish a pipeline of candidates who would not normally consider the sciences as a profession. As such, monetary barriers that limit attendance weaken the potential impact of such programs on populations that these events could potentially serve.

This article highlights a city-wide science festival established at the University of Kentucky College of Pharmacy (UKCOP) titled “*Everything is Science (EiS)*.”^4^ The program was organized to engage the public at restaurants, bars, and other iconic local venues. The festival aimed to close the knowledge gap between scientists and the public with interactive discussions from across scientific disciplines targeting non-scientists and scientists of all ages. The second annual *EiS* festival was held in Lexington, KY, March 4–8, 2019. During the first two years, the festival featured 87 presenters at 20 individual events with more than 350 people in attendance each year. Notably, the festival was organized with a focus on development of a sustainable strategy to engage the public and stimulate learning. After reflection of the first two years, this article provides an overview of the festival, what we achieved, unexpected lessons, and how others can adopt this approach in their cities.

## METHODS

### Everything Is Science Festival (EiS) Overview

*Everything is Science* is a city-wide festival held in Lexington, KY at restaurants, bars and other venues organized by UKCOP. The first annual festival was held April 26–28, 2018 and consisted of 54 presenters at 10 events. The second annual festival was held March 4–8, 2019 and consisted of 33 presenters at 10 events.

### Festival Planning Committee

The planning committee consisted of two faculty from UKCOP, a post-doctoral fellow, and a group of graduate and professional students, along with the College’s Director of Marketing. The first year of the festival also included members from the community, but scheduling conflicts limited the feasibility of their engagement during the planning stage. Each member of the committee was self-selected based on their interest in outreach and science communication. Planning commenced 5–6 months prior to the festival and consisted of weekly 1-h meetings. Meetings consisted of brainstorming and group decisions, and specific tasks were charged to committee members. In the first year, the planning committee identified all speakers and venues. In the second year, session coordinators were tasked with identifying speakers for their event, while the planning committee identified venues and managed logistics for everyone. In the second year, a total of 15 h of programming were planned over five nights.

### Festival Logistics

Speakers were tasked with delivering an engaging talk of ~15–20 min in length to introduce the background and science of their profession with 10–15 min of audience questions and discussion at a local venue while using props or demonstrations (and without PowerPoint slides). Most speakers interspersed their demonstrations, activities, and dialogue with the audience throughout the presentation to provide additional clarity to the science of their profession. Speakers were identified based on how they aligned with the nightly topics and their ability and eagerness to give an engaging and interactive presentation without slides. The most successful presenters were dynamic speakers and could interact with the audience and distill their science down to the lay audience in an easily digestible manner. Those that failed to connect with the audience or fail to relate their science to the audience’s lives were less well-received.

Venues were selected based on their location, availability, and willingness to provide event space for free. Although year one required $2000 for a sound system and other rental items, a cost-neutral event was achieved in year two using strategic community partnerships. We worked with venues that provided space at no cost, but attendees purchased food and drinks to make this partnership worthwhile for the venues on nights that were typically less crowded. We also utilized a portable public announcement (PA) system available from UK’s School of Music. The UK College of Design also provided graphics free-of-charge to advertise the festival. Publicity was disseminated around town through advertising, a press release,^5^ social media, VisitLex (the Lexington Visitor Center), AAAS^6^, the *Behind the Blue* podcast^7^ and on local NBC and ABC affiliate television programs^8^.

### Festival Attendance and Feedback

Attendance at each event was determined by counting the number of individuals at each talk. The fluidity of attendees and audience dynamics in public spaces made it difficult to gain an accurate number at some venues and approximate numbers are given. During year two, comment cards made available to all attendees were used to ask participants to rank their overall experience, venue, and speakers using a Likert scale (1 = Poor; 2 = Fair; 3 = Average; 4 = Good; 5 = Excellent). Attendees were also asked if they would recommend the festival to friends (Yes or No), and space was provided to recommend speakers, topics and additional comments. Recommendations for future topics and speakers were recorded for planning in subsequent years and comments regarding the event were categorized at a high level (e.g., positive, neutral, negative). Comments, not including topic and speaker recommendations, were then entered into a text analyzer (textalyser.net) and top themes identified (e.g., great, speakers, variety, audience), which were then used to categorize all comments.

At the conclusion of the festival nine session coordinators (one coordinator was responsible for creating the survey) were asked to provide assessments using Qualtrics survey using a Likert scale (1 = Strongly disagree; 2 = Disagree; 3 = Undecided; 4 = Agree; 5 = Strongly agree) for the following statements: *My experience was positive; The venue was ideal; The festival improved science literacy; I met new colleagues from UK; I met new people from the community; I had appropriate guidance from the organizing committee; I view the UKCOP more positively because of EiS.* Analysis of data collected was performed with Prism 8.1.1 (GraphPad Software Inc.).

## RESULTS

### The Everything Is Science Festival

**FIGURE 1 |:**
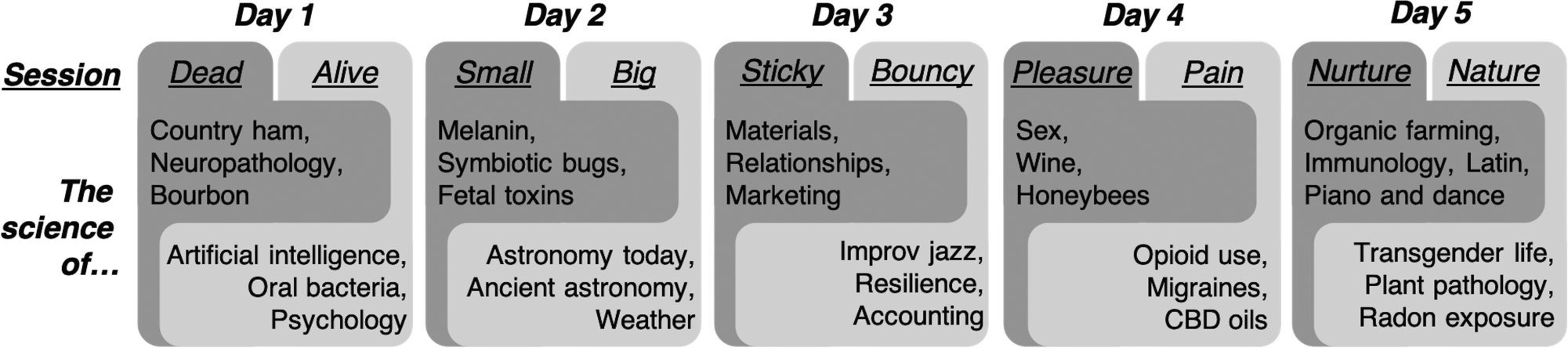
Overview of Programming in Year Two of *EiS* festival. A total of 33 presenters over 5 days at two events per night provided presentations to the community related to the topics shown. Each topic aligned with the broadly defined categories.

Topics covered during the first year of the *EiS* festival focused on the science of Kentucky and were largely centered on healthcare. Having events focused on topics highly relevant to the daily life of people in Lexington was critical to the design and success of the *EiS* festival. Audience interest at most sessions appeared to align with the focus of the session, resulting in an observed lack of “scientific cross-pollination.” During the second year of the festival, a concerted effort was made to engage a more scientifically diverse audience. The planning committee recruited session coordinators from across UK’s campus to help identify speakers, which enhanced the network of potential speakers outside the scope of the planning committee. This change also significantly reduced the time burden on the planning committee. A total of 33 presenters spoke about the science behind their profession, ranging from researchers studying the opioid crisis to an improvisational jazz trio, and a TV meteorologist (Figure 1). Each speaker was allotted 30 min for a 15–20 min talk and 10–15 min of audience engagement. The most engaging speakers blended their talk with audience engagement and encouraged questions throughout. In total, four universities and nine businesses/organizations were represented along with 17 different colleges/units from UK. Broad categories enabled coordinators to be creative about the speakers that were invited. Some coordinators selected their speakers based on their well-established network, while others sought presenters with specific expertise. Both strategies proved successful at bringing engaging speakers to present. Each session ranged from 25 to 60 attendees, with a mixture of people from UK and the community.

### Nightly Topics

The theme for the 2019 *EiS* festival was “Opposites Attract” and each night consisted of two sessions that ran from 5:30 to 7:00 p.m. and 7:30 to 9:00 p.m. at a second location. The topics for each night (i.e., live/dead; sticky/bouncy, etc.) (Figure 1) were selected to attract the broader community with thought provoking titles, while balancing abstract and concrete ideas as well as arts and sciences. This approach also promoted a cross-pollination of scientific concepts to encourage new collaborative efforts across campus and within the community. In fact, local breweries have prepared special releases in honor of the festival themes. This level of engagement is also reflected in the increased number of attendees at each session from year to year. While the coordinators capture quantitative measures of the festival from the number of speakers and number of attendees there has not been an effort to capture the organic formation of new collaborations in real time, which would detract from the true intention of the event. However, feedback is requested from all attendees to identify any major issues with various aspects of the festival.

### Audience Feedback

**FIGURE 2 |:**
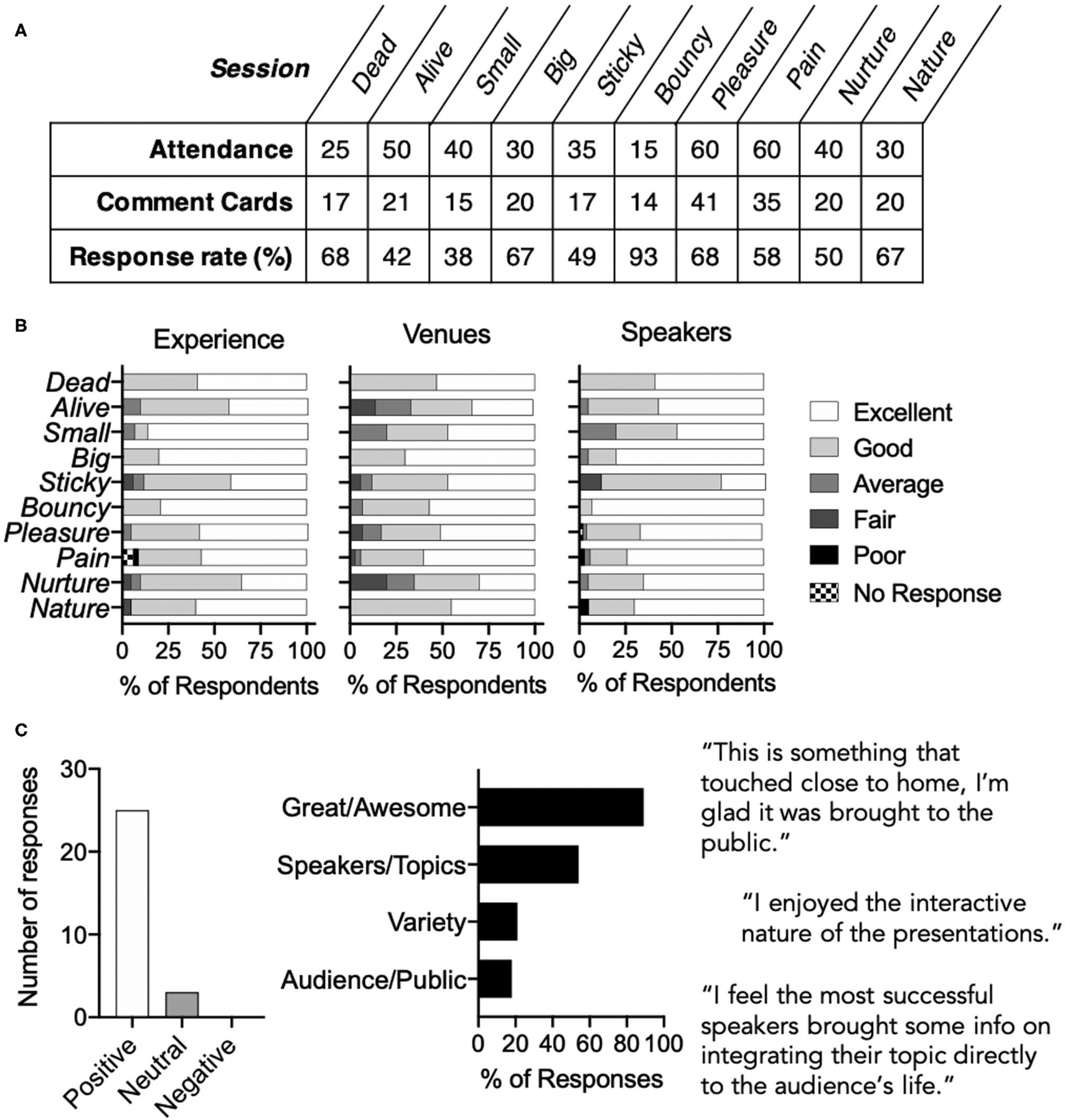
Feedback Assessed During the Second Year of the *EiS* festival. Assessments were made using comment cards available to attendees at each of ten sessions. **(A)** Attendance and comment card response rates. **(B)** Attendee rankings for experience, venue, and speakers. **(C)** Qualitative analysis of 28 audience comments by tone and thematic classification with selected quotes highlighted.

Comment cards provided to all attendees were collected to assess the quality of the venue, speakers, experience and offering space to recommend future speakers, topics, and open-ended comment space. Altogether, 220 comment cards were returned for a 63% response rate (Figure 2A). In total, the festival was highly rated with the majority of attendees ranking each of the categories as “Good” or “Excellent” (Figure 2B). The overall experience received a rating of 4.5 ± 0.7 with 94% of respondents scoring their experience as “Good” or better. Similar data were obtained for the venues, with an overall rating of 4.3 ± 0.8, with 86% of attendees rating the venues as “Good” or better. Notably, two sessions, “*Alive*” and “*Nurture*” were in venues that were excellent for attracting the public and resulted in a noisy event, which was the most common feedback received on the comment cards. Speakers received high marks with a ranking of 4.6 ± 0.7 and 95% of respondents ranking the speakers as “Good” or better. Finally, 99% of respondents said they would recommend the festival to a friend, while 3 people (1%) did not respond. There were an additional 28 comments in the open-ended question section regarding the events (Figure 2C) with 89% positive (25 out of 28) and 11% neutral (3 out of 28). The three neutral comments included recommendations or observations from the event. When analyzed by topic, 89% offered praise for the festival, 54% commented on the speakers and topics, 21% commented on the variety of the topics, and 18% commented on the benefit to the audience/public.

Of the 220 comment cards received, a total of 71 recommendations for future sessions were provided (Table 1). Some recommendations for topics had been previously discussed during the 2018 EiS Festival, while others were in included in the same year that the topic recommendations were received. Importantly, topic recommendations were used in planning the *EiS* festival held in 2020. A number of additional recommendations from the audience have not been included in an *EiS* Festival thus far, and provides a breadth of topics for future planning.

### Session Coordinator Feedback

Post-festival surveys completed by the session coordinators offered a similar story (data not shown), with 100% of coordinators responding with “Agree” or “Strongly Agree” that the experience was positive. Two coordinators were unhappy with the venues, which aligns with attendee feedback. Notably, coordinators used different mechanisms to identify speakers and seven of nine coordinators met new colleagues at UK and new people from the community, while the other two coordinators identified speakers within their network. Additionally, eight out of nine coordinators view UKCOP more positively after helping to coordinate their event, while one coordinator was undecided.

## DISCUSSION

As we reflect upon the first two years of the *EiS* festival, we consider our achievements and shortcomings in an effort to improve upon future events and provide a framework to continue these efforts in other cities. Educational outreach programs established at institutions of higher education, especially research-intensive institutions, generally align with extramural funding cycles and often end when a specific grant expires. Novel, low-cost strategies are necessary to recognize the benefits of such programs, while breaking away from the funding cycle paradigm of many outreach efforts. Through the *EiS* festival, we established a paradigm for cost-neutral planning, which eliminates the need to identify funding sources.

The vibrant atmosphere of Lexington and UK offers a platform to support the *EiS* festival. The success of the festival is largely attributed to the selection of premier speakers from across UK’s campus and the Commonwealth of Kentucky. Engagement between the university and the city provides an ideal opportunity for the community to attend events and learn about the science around them. Importantly, collegiate administrators at UK are supportive of these types of endeavors and find them to be beneficial to multiple constituents (i.e., students, faculty, college, university, city, and Commonwealth). Support for the *EiS* festival did not stop at the College level as representatives for the University President, Lexington Mayor, and others expressed interest in the need for opportunities to engage the public and highlight cutting-edge science being performed across the Commonwealth. While the *EiS* festival is organized for people of all ages to enjoy, the audience has been mostly college-aged and older, with children present at only a few events. While strategies to increase attendance of different ages may be considered in future years, it is notable that other outreach events in Lexington target elementary school students or college students only. Our selection as pubs and bars is most likely the reason for the older skewing crowd, even though all venues are open to all ages for the events. Notably, the venues continue to serve as key partners and have been eager to continue working with the *EiS* festival due to the revenue generated from food and drinks on nights that would not normally attract a crowd.

Audience engagement was a key design principle of the festival by tasking presenters with using hands-on activities and engaging demonstrations. Each speaker used their time differently to engage the audience with question/answer and interactive components throughout the talk, or a 10–15 min talk followed by a hands-on/demonstration session. It should be noted that the *EiS* festival has been fortunate in the quality of the speakers, but not all speakers hit the mark on providing an engaging talk. Comments provided from the audience indicate that the most successful speakers relate their science to the lives of the audience so that they could more easily engage with the content of the presentation and discussion. Additionally, the willingness and eagerness of a speaker to work within the confines of a slide-free presentation also has some indication on who will embrace the task in a meaningful way. Importantly, strategies used for speaker recruitment could be one explanation for the success of most speakers given that most speakers were identified from a friend/colleague network of people who align with a specific topic and are identified by the coordinator to be a dynamic presenter. However, other speakers were recruited based solely on the scientific topic regardless of speaking attributes. Given the importance of the network for not only identifying speakers, but also advertising, the *EiS* festival continues to work with other outreach programs on campus to synergize activities and increase the speaker pools and target audience. By enhancing the speaker pool and building the festival around audience recommended topics (Tables 1, 2) we will be able to continue to ensure that the audience connects with the content and engages with the science and the speakers in a meaningful way.

Outside of the societal impact, students on the planning committee also benefited from helping to coordinate the festival (Leshner, 2007). Graduate and professional students, as well as faculty and administrators, are often unaware of the potential impact of outreach programs and similarly unfamiliar with the effort necessary to pull together a program like *EiS* (Andrews et al., 2005; Ecklund et al., 2012). As we focus on training students to practice and work at the top of their profession, we must provide opportunities for students to engage in organizing outreach events and as a means to develop “soft skills” (O’Connor et al., 2011; Laursen et al., 2012; Banerjee, 2016; Clark et al., 2016). Co-curricular training in public engagement, outreach, event organization, and promotion provide critical experiences. These opportunities result in well-balanced students and future leaders who realize the impact of their efforts and will, ideally, continue to pursue similar efforts throughout their careers. While opportunities for such events do not always present themselves, when opportunities arise, trainees inclined toward such an experience are able to grow as students and professionals, to better understand their capacity as community leaders.

One unexpected outcome of the festival is the number of speakers who developed novel teaching strategies to convey their science. The information-deficit model aims to deliver science to an ignorant audience and efforts to fill this “ignorance” gap include introduction of open-access data and community presentations that take the tact of “the data speaks for itself.” However, these strategies have not been as successful as originally lauded (Sturgis and Allum, 2004; Simis et al., 2016). The limitations around this strategy have encouraged new approaches to connect with communities (Killikelly, 2018). We found that by restricting presentations to props and demonstrations, speakers were inclined to think more creatively about the analogies used to describe their science and interact with the audience. Several speakers offered that they normally utilize PowerPoint or internet-based content to describe difficult concepts, but the *EiS* festival encouraged them to develop innovative teaching strategies that get the audience out of their seats to interact with the science rather than be an observer, which they will adopt in the classroom. Examples of some teaching strategies are shown in Table 2. An example of one teaching tool that was well-received and relevant to the audience was focused on cardiovascular disease. The talk presented by a cardiologist and cardiovascular research team focused on cardiac health and conditioning brought a handheld echocardiograph to a café to demonstrate cardiac output through live imaging of a volunteer. The relevance of cardiovascular disease to the population of Kentucky provided an opportunity to not only visualize what procedures are being done on patients in the clinic, but also provided an opportunity for the audience to discuss heart health in an informal setting and ask questions of a physician outside of the clinical setting.

Of importance to the design of the festival is also the aspect of inclusivity and diversity to promote and highlight the science of female and underrepresented minority speakers. The importance of diversity in science is well-documented (Marx and Roman, 2002; Stout et al., 2011), and the representation of speakers in events like this has been shown to positively impact recruitment into STEAM fields (Museus et al., 2011). Given that our goal is to extend public outreach to the Lexington community we aim to exhibit representation that aligns with the demographics of our community, which is 50% female and 20% underrepresented minority (URM) according to the US Census Bureau^9^. While diversity extends far beyond the gender binary and racial lines into socioeconomic status, religion and ethnicity, we do not collect demographic data on those affiliated with the *EiS* festival and cannot properly assess the true diversity of our speakers. Nevertheless, we have made a concerted effort to improve upon our diversity by inviting diverse voices to participate in the festival and we have worked with the UK Office of Institutional Diversity to ensure that recruitment is inclusive. Notably, when session coordinators are responsible for identifying speakers, the planning committee is unable to direct who presents in each session, regardless of how much the importance of diversity is impressed upon the session coordinators. Therefore, it is important to ensure that there is a diversity of backgrounds on the planning committee and amongst session coordinators. During the first three years of the festival we have maintained a 50% male/female ratio and have increased the URM representation from 5% in the first two years to 20% in year 3, which closely resembles the demographics of the community according to these two metrics. Additionally, the *EiS* festival exceeds the representation amongst faculty at UK (female: 42%, URM, 8%). It is also unclear what the true diversity of the audience is, but determining age, profession, affiliation and who is a new attendee (Kennedy et al., 2017), should be considered as we move forward to enhance the impact of the festival as it continues to grow. We hope that this festival will continue to provide the community with scientific role models that represent the community to serve as a platform and a strategy to break down the barriers of representation in STEAM fields.

Lessons garnered from 3 years of experience also provided guidelines to facilitate adoption of similar festivals in other cities. The venues, speakers, and topics described here are specific to the *EiS* festival held in Lexington, KY, but the overall paradigm established by our team can be disseminated around the world to improve public health and science literacy and as a means to recruit future scientists and practitioners. The most important lessons learned from our experience thus far are: (1) start planning early; (2) diversify events with broad topics; (3) keep events free to make science accessible to everyone; (4) enhance engagement with speakers who use activities and demonstrations that make science relevant to the audience; (5) utilize session coordinators to enhance speaker networks; and (6) have fun. The overview and tips highlighted here are selected as the most significant lessons learned thus far. As planning continues for future events, new hurdles, lessons and successes will surface. Although educational opportunities, such as these, require significant effort to launch, sustainable strategies are important to consider with the goal of continuing to advance scientific literacy.

In conclusion, the *EiS* festival serves as a no-cost method to engage with the community to improve science literacy with strong support from all constituents to continue these offerings in future years using a similar format.

## Figures and Tables

**TABLE 1 |:** Overlap between audience recommendations from the 2019 EiS festival and the first three years of EiS festivals.

EiS Festival	Topics
2018	Addiction • Biomedical science • Cancer • Cardiology • Craft beer • Illegal drugs • LGBTQ • Mental health • Oncology • Sex • Nanoparticles
2019	Addiction • Artificial intelligence • Bacteria • Biomedical science • Child psychology • Cognitive science • Craft beer • Food LGBTQ • Mental health • Sex • Space • Nanoparticles • Nanosurfaces
2020	Addiction • Ancient world • Art • Biomedical science • Cardiology • Climate change • Diversity • Ecology • Environmental issues • Genetic mutations • Global warming • LGBTQ • Mating • Mental health • Nutrition and vitamins • Sex • Storytelling
Not yet included in EiS Festival	ADHD • Allergies • Automotive and transportation industry • Bio-hacking • Bugs impact on disease • Cheese • Computer game playing • Dogs • Evolutionary biology • Fire dynamics • Genetically modified organisms • Hepatitis • Incarceration • Longevity • Mine restoration • Ocean depths • Parenting • Pharma drug approval process • Politics • Prosthetic limbs • Quantum mechanics • Robotics • Sensory processing disorders • Sleep • Social media • Spontaneous combustion • Survival of the fittest • Thermodynamics

**TABLE 2 |:** Teaching tools established by speakers at the *EiS* festival.

Speaker Affiliation	Teaching tool
2nd Grade STEM teacher	*Curiosity*: Write a question about ice cream - curiosity starts early
Alltech Inc.	*Hydrophobicity:* Magic milk with food coloring and soap to explain water and fat interactions
UK Healthcare	*Heart function:* Live demo of normal heart with a handheld echocardiograph
UK College of Design	*Architectural Design:* Described redesign of the Kentucky Native Cafe….in the cafe
McCauley Bros. feed store	*Horse Nutrition:* Full size digestive track demo made from household items
UK College of Pharmacy	*B cell immunology:* Tennis balls with screws to describe B cell specificity
UK College of Pharmacy	*Antibiotic resistance:* Burglars (antibiotics) use different ways to break into houses (bacteria), but houses have many locks
Food Writer	*Food science*: Ham samples to taste
UK Healthcare	*Neuropathology:* A human brain and tissue sections
UK Engineering	*Microbiome*: Oral bacterial samples
NBC-affiliate LEX18-TV	*Weather patterns:* Highs/low atmospheric pressures portrayed by two attendees
Eastern Kentucky University	*Astronomical activity*: The sun (student in a chair) is stationary, while planets (three students) revolve around the sun at their own pace Watch for the planetary alignment!
UK Arts & Science	*Improvisational jazz and dance*: A pianist, trombonist and dancer improv and discuss their thought process
UK College of Agriculture, Food and Environment	*Food supply threats:* Pizza assembly while describing the plant pathogens that threaten all nine ingredients

## Footnotes

1 Science Cafés: sciencecafes.org (accessed July 04, 2020).

2 Café Scientifique: caféscientifique.org (accessed July 04, 2020).

3 EvalFest: evalfest.org (accessed July 04, 2020).

4 *EiS* festival website: http://eis.createuky.net (accessed August 19, 2020).

5 Press release: (2018) https://uknow.uky.edu/arts-culture/everything-science-University-kentucky-hosts-city-wide-science-festival; (2019) https://uknow.uky.edu/uk-happenings/opposites-attract-uk-s-everything-science-festival (accessed July 04, 2020).

6 American Association for the Advancement of Science (AAAS) publicity: https://www.aaas.org/event/everything-science-festival-lexington-ky (accessed July 04, 2020).

7 Podcast: (2019) http://behindtheblue.libsyn.com/february-28-2019-everything-is-science (accessed July 04, 2020).

8 Local TV shows: (2018) https://www.youtube.com/watch?v=AbUsNSxeC1k and http://www.clipsyndicate.com/video/play/7345598/everything_is_science. (2019) https://www.wtvq.com/2019/02/27/everything-science-festival/ (accessed July 04, 2020).

9 US Census Bureau (Lexington-Fayette, KY) webpage: https://www.census.gov/quickfacts/lexingtonfayettekentucky (accessed July 04, 2020).
